# Why patient safety is akin to weaving textiles: an International Society for Quality in Health Care perspective on the 7th Global Ministerial Summit on Patient Safety 2025

**DOI:** 10.1093/intqhc/mzaf072

**Published:** 2025-08-12

**Authors:** Jeffrey Braithwaite, Anuradha Pichumani, Shin Ushiro, Leslee Thompson, Peter Hibbert, Ezequiel Garcia Elorrio

**Affiliations:** Centre for Healthcare Resilience and Implementation Science, Australian Institute of Health Innovation, Faculty of Medicine, Health and Human Sciences, Macquarie University, Sydney, Australia; International Society for Quality in Health Care (ISQua), Dublin, Ireland; Executive Director, Sree Renga Hospital, Chengalpattu, Tamil Nādu, India; Deputy Hospital Director and Chief Patient Safety Officer, Kyushu University Hospital, Japan; CEO, Health Standards Organization (HSO) and Accreditation Canada, ON, Canada; Centre for Healthcare Resilience and Implementation Science, Australian Institute of Health Innovation, Faculty of Medicine, Health and Human Sciences, Macquarie University, Sydney, Australia; International Society for Quality in Health Care (ISQua), Dublin, Ireland; Director of Health Care Quality and Patient Safety, Institute for Clinical Effectiveness and Health Policy, Buenos Aires, Argentina

**Keywords:** patient safety, improvement science, person centred care, quality improvement, resilience healthcare systems, systems science

## Abstract

The 7th Global Ministerial Summit on Patient Safety, convened in Manila in April 2025, advanced global efforts to reduce harm in healthcare under the theme ‘Weaving Strengths for the Future of Patient Safety’. Drawing on the metaphor of textile weaving, the Summit emphasised that safeguarding patients is a collaborative endeavour, requiring integrated action across sectors and systems. The Mandaluyong Declaration reaffirmed commitments to the WHO Global Patient Safety Action Plan (2021–2030) and made four pledges: strengthening global collaboration, advancing leadership and governance, integrating patient safety into disaster preparedness and climate resilience, and building people-centred safety systems. The mission of the International Society for Quality in Health Care (ISQua), a key presence at the Summit, is aligned with these aims, contributing through its White Paper on patient safety, its Green Paper on climate-resilient health systems, and its Person-Centred Care White Paper. The Summit marked a shift from commitment to implementation, recognising the importance of climate change, digital transformation, and equity as integral to safe care. Patient safety now stands as both a global health and environmental priority. ISQua will continue supporting this work through advocacy, standards development, and partnerships, helping to weave patient safety into the daily practice of healthcare worldwide.

## Background

The 7th Global Ministerial Summit on Patient Safety was held in Manila, The Philippines from 3 to 4 April 2025. The theme this year was ‘*Weaving Strengths for the Future of Patient Safety Throughout the Healthcare Continuum*’. This concept was an inspired choice and reflects the requirement—in both weaving textiles and keeping patients safe—to collaborate. No one can create a fine Turkish Ghiordes rug or keep a patient safe on their own.

As with recent summits (Table 1), the participating health ministers of the world endorsed a communiqué. This was released by our Filipino hosts at the end of the proceedings.

**Table 1.** Previous patient safety ministerial summits

Meeting, year	Theme, communiqué, link	Key issues, ideas and recommendations
London, England, 9–10 March 2016	‘Inaugural meeting: The Patient Safety Global Action Summit 2016’ **Overarching theme: toward a culture of safety and learning** https://www.who.int/news-room/events/detail/2016/03/09/default-calendar/first-patient-safety-global-action-­summit-2016	Four expert panels: System-based approaches to patient safety. Emerging complexities in healthcare delivery. Innovative tools for safety, including digital health and behavioural insights. Learning from other industries and countries. WHO’s Director-General outlined five action points: Political commitment and leadership. Policies that support safety improvements. Paradigm shift to encourage open reporting. Performance measurement through data and benchmarking. Patient safety movement: A global call to action.
Bonn, Germany, 29–30 March 2017	‘Second Global Ministerial Summit on Patient Safety 2017’ https://www.bundesgesundheitsministerium.de/fileadmin/Dateien/3_Downloads/P/Patientensicherheit/Patient_Safety_Summit_2017_Final_Report.pdf	The Summit endorsed: 1. Political commitment and leadership 2. Global collaboration 3. Call for a WHA resolution to: Launch a Global Action on Patient Safety and Establish September 17 as World Patient Safety Day. 4. Core political messages to ministers Patient safety must be a core value in all health systems. Transparency, accountability, and learning from errors are vital. Investment in safety is cost-effective and improves outcomes. 5. Workshops and thematic focus: key topics: Economics of patient safety. Digital health and mobile technologies. Infection prevention and control. Global learning systems for safety improvement. 6. Science and practice recommendations of experts, who called for: Better data systems to track and learn from adverse events. Education and training for healthcare professionals. Patient engagement in safety initiatives.
Tokyo, Japan, 13–14 April 2018	‘Third Global Ministerial Summit on Patient Safety 2018’ **The Tokyo Declaration on Patient Safety** https://www.mhlw.go.jp/file/06-Seisakujouhou-10800000-Iseikyoku/0000204005.pdf	1. The Tokyo Declaration Calls for global action to reduce avoidable harm in healthcare by 2030. Emphasizes leadership, collaboration, and accountability at all levels—­local, national, and global. 2. Patient safety as a foundation of Universal Health Coverage (UHC) 3. Evidence-based policy and practice urging countries to: Develop national patient safety strategies. Invest in data systems to monitor harm. Promote research and innovation in safety. 4. Learning from other sectors especially aviation, nuclear, and other high-reliability industries 5. Empowering patients and health workers Patients should be active partners in safety. Health workers need training, support, and a just culture to report and learn from errors. 6. Global collaboration
Jeddah, the Kingdom of Saudi Arabia, 2–3 March, 2019	‘Fourth Global Ministerial Summit on Patient Safety 2019’. **The Jeddah Declaration on Patient Safety** https://www.spsc.gov.sa/English/Summit/Documents/%D8%A7%D9%84%D9%85%D9%88%D9%82%D8%B9.p	1. Patient safety as a global health priority Reinforced the idea that unsafe care is a major global health burden, especially in low- and middle-income countries. Emphasized the need for system-wide safety improvements and leadership commitment. 2. Digital health and innovation Highlighted the role of digital technologies in improving safety, 3. Patient and family engagement Advocated for empowering patients and families as active partners in care. Encouraged transparent communication and shared decision-making. 4. Workforce safety and culture Stressed the importance of a just culture. Promoted training, well-being, and resilience of staff as essential to patient safety. 5. Global collaboration and learning Called for international cooperation to share best practices and data. Supported the development of global learning systems and standardized metrics. 6. Economic case for safety Demonstrated that investing in safety saves lives and reduces costs. Cited the high economic burden of preventable harm and medical errors.
Montreux, Switzerland, 23–24 February 2023	‘Fifth Global Ministerial Summit on Patient Safety 2023’ **The Montreux Charter on Patient Safety –‘Less Harm, Better Care—from Resolution to Implementation’** https://pss2023.ch/wp-content/uploads/2023/03/Montreux_Charter_Patient_Safety_Summit_2023.pdf	1. Patient safety as a fundamental right Declares that safe healthcare is a human right. Calls for zero avoidable harm in healthcare systems globally. 2. Political leadership and accountability Urges governments and health leaders to prioritize patient safety in national health agendas. Emphasizes transparency, accountability, and governance. 3. Empowering patients and families Patients should be active partners in their care. Promotes shared decision-making, open communication, and respect for patient voices. 4. Workforce safety and culture Advocates for a just and safe culture for healthcare workers. Calls for training, support, and protection of staff to ensure they can deliver safe care. 5. Data, learning, and innovation Encourages the use of data and digital tools to detect, learn from, and prevent harm. Supports research, innovation, and global knowledge sharing. 6. Equity and inclusion Recognizes that inequities in care contribute to unsafe outcomes. Calls for inclusive policies that address disparities in healthcare access and quality. 7. Global collaboration Reinforces the need for international cooperation to build resilient, learning health systems. Aligns with WHO’s Global Patient Safety Action Plan 2021–2030.
Santiago, Chile, 17–18 April 2024	‘Sixth Global Ministerial Summit on Patient Safety 2024 “Bringing and sustaining changes in patient safety policies and practices”’ **The Santiago Commitment Charter** https://psschile.minsal.cl/	1. Focus on implementation and sustainability The Summit emphasized how countries are implementing and sustaining patient safety strategies. It aimed to translate lessons learned into national commitments and concrete actions. 2. Alignment with WHO’s Global Patient Safety Action Plan (2021–2030) Discussions were framed around the seven strategic objectives of the WHO Action Plan. Countries shared experiences on how they’ve adapted these objectives to their local contexts. 3. Key areas of focus Safety culture in healthcare institutions. Patient and family empowerment. Safety in specific care processes. Resilience of health systems in uncertain scenarios (e.g. pandemics). Education, communication, and training of healthcare teams. Quality assurance systems. Use of digital health and AI to enhance safety. 4. Global collaboration and knowledge exchange The Summit served as a platform for sharing innovations, challenges, and successes. It reinforced the importance of international cooperation in advancing patient safety.
Manila, Philippines, 2–4 April 2025	‘Seventh Global Ministerial Summit on Patient Safety 2025’ **The Mandaluyong Declaration: Weaving Strengths for the Future of Patient Safety Throughout the Healthcare Continuum** https://www.who.int/news/item/04-04-2025-the-7th-global-ministerial-summit-on-patient-safety-2025 FinalDraftofMandaluyongDeclaration_asofMarch272025(1) (5).pdf	1. Accelerating implementation of the WHO Global Patient Safety Action Plan (2021–2030) Emphasis on measurable progress in implementing the seven strategic objectives. Focus on national accountability and policy integration. 2. Digital transformation and AI in patient safety Exploration of AI-driven tools, predictive analytics, and digital platforms to reduce harm. Addressing ethical and safety concerns in digital health. 3. Equity and inclusion Ensuring safe care for all, especially marginalized and vulnerable populations. Tackling disparities in safety outcomes across regions and demographics. 4. Resilient health systems Strengthening systems to withstand emergencies and disruptions (e.g. pandemics, climate events). Promoting learning health systems that adapt and evolve. 5. Patient and workforce empowerment Reinforcing the role of patients as partners in safety. Supporting healthcare workers’ well-being, training, and safety culture. 6. Global collaboration and leadership Renewed calls for international cooperation, data sharing, and joint research. Possibly reaffirming or updating the Montreux Charter or similar declarations.

Across the seven Summits, we can see from Table 1 how they have evolved from raising awareness and calling for political commitment to advocating for health systems to implement systemic change and leverage innovation. Each Summit has built upon the last, reinforcing the idea that patient safety needs to be a global, ethical, and strategic priority. We can also observe some discernible trends (Table 2).

**Table 2.** Key trends across the summits

Trend	Early emphasis (2016–2018)	Mid-phase (2019–2023)	Recent focus (2024–2025)
**Political leadership**	Strong foundational theme	Reinforced through charters	Tied to accountability and equity
**Systemic approaches**	Introduced as a core concept	Expanded with digital tools	Embedded in national strategies
**Patient empowerment**	Actionable idea	Central to Montreux Charter	Deepened with co-production models
**Workforce safety**	Limited focus	Gained prominence post-2019	Safety culture integrated with system resilience
**Digital health & AI**	Not as prominent	Introduced and expanded on from 2019	Central in 2025 discussions
**Global collaboration**	Initiated in London	Strengthened in Bonn and Jeddah	Institutionalized in Chile & beyond with the Global Patient Safety Action Plan
**Equity and inclusion**	Needs more attention	Recognized	Explicitly prioritized in 2025
**Climate change and sustainability**	Awareness	Raised during the COVID pandemic years	Given much prominence in 2025
**Patient safety as a right**	Implicit	Explicitly recognised	Expanded and linked to equity

## The Mandaluyong Declaration

Having established discernible Summit trends, we can turn to the Filipino Summit. Much of what has been documented in the first six events has been absorbed into patient safety thinking and agreed amongst patient safety advocates, researchers, policymakers and clinicians over the years. But what about the seventh Summit, and its likely contribution over time? We turn to a brief exegesis of its highlights and potential influence.

This Mandaluyong Declaration on Patient Safety explicitly indicates that it builds on the work of the six previous Summits and reaffirms an ‘*unwavering determination to make patient safety a universal imperative*’. In keeping with the theme, the Declaration opens with the words ‘*We, the Patient Safety Weavers* …’. It links its commitments to patient safety to the World Health Organization (WHO) Assembly’s resolution 72.6 and endorses the WHO’s Global Patient Safety Action Plan 2021-2030, both powerful statements of intent, but asks for ‘*renewed urgency*’. The Declaration supports ‘*patient safety as a foundational pillar of resilient, people-centred and equitable health systems*’. Participants made four pledges in the Declaration (Table 3); all resonate with the International Society for Quality in Health Care’s (ISQua’s) mission.

**Table 3.** The four pledges of the Mandaluyong Declaration and ISQua’s work in support

Pledge	Summary	ISQua contribution to this pledge
**1. Strengthen Global Collaboration for Patient Safety**	Together, inspired by *bayanihan* (Filipino for solidarity and collectivism), the aim should be to improve patient safety globally through transparency, mutual accountability, and shared learning	*Safe Care is the Right Care: The ISQua White Paper on Patient Safety for Healthcare Organisations*, aligning ISQua’s work with WHO’s *Global Patient Safety Action Plan 2021–2030*, also resonates with Pledges #1 and #2, and was officially released at the Manila Summit [1]
**2. Advance Leadership and Governance in Patient Safety**	Prioritise patient safety in health governance, invest in primary care, and strengthen leadership to drive accountability and continuous improvement	
**3. Integrate Patient Safety into Disaster and Emergency Preparedness and Climate Resilience**	Integrate patient safety in disaster plans, mitigate risks, ensure care continuity, and build resilient health systems amid climate change impacts	Green Paper on climate change (*Green Care is High Quality Care: The ISQua Green Paper and Call to Action for Environmentally Sustainable and Climate Resilient Health Systems*) [2]
**4. Build a People-Centred Approach to Patient Safety**	Value experiences and expertise, strengthen inclusive decision-making, and co-create solutions for a people-centred, equitable, and sustainable patient safety future	ISQua White Paper, *Person-Centred Care Systems: From Theory to Practice* [3]

So we can see that this meeting did not so much introduce new ideas as consolidate thinking and argue for more effort, ingenuity and collaboration. To give that effect, the Declaration concludes with a *Call to Action*. This commits the weavers to enhancing patient safety through leadership, evidence-based frameworks, global partnerships, workforce investments, technology, and stakeholder engagement, urging all to join in embedding safety principles into health systems.

In formulating the Declaration, the ministers not only fully endorsed it but also recognised the environmental agenda. This is an important pivot: the sustainability idea was suggested in Summits 5 and 6, but now it becomes a part of the patient safety armamentarium. The ministers argued that climate change introduces new risks for patients, for example, from floods, droughts, wildfires and rising temperatures, as well as stimulating changes to the patterns of vector-borne diseases [4–6]. These stand to further jeopardise patient safety around the globe.

## How we are supporting the Declaration and its pledges

The authors of this update are board members, members and fellows of ISQua. ISQua has support to offer our Summit colleagues, the ministers of the world, and the WHO in furtherance of the Mandaluyong Declaration. For us, too, patient safety should be woven into the very fabric of care. In fact, ISQua members will find familiarity with this idea: the ISQua 2018 Conference in Kuala Lumpur, Malaysia, had as its theme *Heads, Hearts and Hands: Weaving the Fabric of Quality and Safety.*

Building on this concept, we were very taken during the Manila Summit by the tradition of *bayanihan*. This Filipino notion of collective responsibility and community spirit infused presentations and discussions. This resonates clearly with ISQua’s mission of togetherness in quality and safety through knowledge, network, voice, and action.

Historically, and in keeping with the tenor of the Summit, ISQua has made many contributions to patient safety. For example, as we show in Table 3, the ISQua-WHO Task Force on Patient Safety was formed in response to the WHO’s Global Patient Safety Action Plan, leading to a white paper on patient safety. *Safe Care is the Right Care: The ISQua White Paper on Patient Safety for Healthcare Organisations* resonates with Pledges #1 and 2, and was officially released at the Manila Summit [1]. An earlier ISQua White Paper, *Person-Centred Care Systems: From Theory to Practice* [3], is synergistic with the Declaration’s Pledge #4. And ISQua’s celebrated Green Paper on climate change (*Green Care is High Quality Care: The ISQua Green Paper and Call to Action for Environmentally Sustainable and Climate Resilient Health Systems*) [2] fits squarely with Pledge #3.

That third paper is newer to patient safety thinking, and it makes three points which act to strongly support the Mandaluyong Declaration’s Pledge #3: First, the climate challenges facing healthcare systems are growing more intensive; second, everyone has a duty to be educated about climate change, ecological disruption, and climate-induced risks to good health; and third, we each have a responsibility to build climate-resilient, environmentally sustainable, and low-carbon health systems.

The engine room of ISQua is its institutional members, individual members, Fellows and the countries in which we operate—over 70 at last count. Helping provide safer care, quality services and continuous improvement is our core business. ISQua’s steadfast commitment to the safety endeavour aims to support, in complete agreement with the Filipino Summit’s Declaration, the estimated 421 million patients who are hospitalised annually and the billions who see a practitioner in the community: for example, in family physicians’ offices, baby health clinics, rehabilitation settings, general practices and residential aged care settings. All must receive quality care and be kept safe.

## Conclusion

ISQua stands not only with the health ministers, experts, clinicians, policymakers, patients and supporters who attended the 7^th^ Global Summit, but also with all those who share our values. Not all ministers can attend the Patient Safety Summits, but they—and we—can help spread the messages every day. Indeed, ISQua, a designated non-state actor working with WHO, is fulfilling its mission in conjunction with other international and national bodies, accreditation agencies, governments, ministries and health sectors and systems. After 40 years of international leadership, collaboration and advocacy, ISQua understands deeply that patient safety is a journey. And, as the Mandaluyong Declaration reminds us, weaving safety into the tapestry of healthcare requires the alignment and integration of systems, processes and people.
